# Review on the Current Trends of Toxoplasmosis Serodiagnosis in Humans

**DOI:** 10.3389/fcimb.2020.00204

**Published:** 2020-05-08

**Authors:** Rochelle Haidee D. Ybañez, Adrian P. Ybañez, Yoshifumi Nishikawa

**Affiliations:** ^1^National Research Center for Protozoan Diseases, Obihiro University of Agriculture and Veterinary Medicine, Obihiro, Japan; ^2^Institute of Molecular Parasitology and Protozoan Diseases at Main and College of Veterinary Medicine, Cebu Technological University, Cebu City, Philippines

**Keywords:** *Toxoplasma gondii*, toxoplasmosis, serodiagnosis, recombinant antigens, human

## Abstract

Toxoplasmosis is a widely distributed zoonotic infection caused by the obligate intracellular apicomplexan parasite *Toxoplasma gondii*. It is mainly transmitted through the ingestion of oocysts shed by an infected cat acting as its definitive host. The key to effective control and treatment of toxoplasmosis is prompt and accurate detection of *T. gondii* infection. Several laboratory diagnostic methods have been established, including the most commonly used serological assays such as the dye test (DT), direct or modified agglutination test (DAT/MAT), indirect hemagglutination test (IHA), latex agglutination test (LAT), indirect immunofluorescent test (IFAT), enzyme-linked immunosorbent assays (ELISA), immunochromatographic tests (ICT), and the western blot. Nonetheless, creating specific and reliable approaches for serodiagnosis of *T. gondii* infection, and differentiating between acute and chronic phases of infection remains a challenge. This review provides information on the current trends in the serodiagnosis of human toxoplasmosis. It highlights the advantages of the use of recombinant proteins for serological testing and provides insight into the possible future direction of these methods.

## Introduction

Toxoplasmosis is a widely distributed zoonotic infection caused by the obligate intracellular apicomplexan parasite *Toxoplasma gondii*. *T. gondii* infects humans and almost all warm-blooded animals, making it one of the important parasites affecting public health and animal production. It is mainly transmitted through the ingestion of oocysts shed by an infected cat as its definitive host (Dubey and Beattie, 1988; Dubey and Jones, 2008; Torrey and Yolken, 2013). Toxoplasmosis affects approximately one-third of the world's human population. However, it is generally asymptomatic in immunocompetent individuals, or it may manifest flu-like symptoms and other non-specific clinical signs (Dubey, 1991). The disease may even be severe or fatal in immunocompromized patients (Montoya and Liesenfeld, 2004). Vertical transmission of the parasite through the placenta from the infected mother may compromise the life of the fetus and the infected mothers (Gatkowska et al., 2006; Elmore and Jones, 2010; Sun et al., 2013). The key to effective control and treatment of toxoplasmosis depends on accurate detection of *T. gondii* infection. The utilization of highly sensitive and specific diagnostic methods is a vital step in the prevention and treatment of the disease (Terkawi et al., 2013).

Due to its non-specificity of clinic signs, the diagnosis of *T. gondii* infection cannot be made through the assessment of clinical manifestations (Tenter et al., 2000). *T. gondii* diagnosis for immunocompromized patients is usually done using polymerase chain reaction (PCR), hybridization assays, isolation, and histological analysis. For congenital cases, diagnosis is through direct detection of the organism through mouse inoculation, cell culture or PCR from samples collected from amniotic fluid (Cazenave et al., 1991), cerebrospinal fluid, blood and urine (Fuentes et al., 1996), and through ophthalmologic and radiological examinations (Montoya, 2002; Pomares and Montoya, 2016). However, the most common form of *T. gondii* infection is latent, wherein the parasites are usually not found in the circulation, and isolating the parasites are particularly challenging (Robert-Gangneux and Dardé, 2012). However, as *T. gondii* induces an intense and often persistent humoral immune response with detectable antibody titers, regardless of the clinical manifestations in the infected individuals (Parmley et al., 1992; Dubey, 2008), serological tests that detect specific antibody responses are deemed useful.

Over the years, there have been several serological methods established for the diagnosis of toxoplasmosis, and many have produced satisfactory results. However, the development of specific and reliable approaches for *T. gondii* infection serodiagnosis, which could ideally differentiate between acute and chronic phases of infection, remains very complicated. This review offers updated knowledge on the current trends in human toxoplasmosis serodiagnosis. It emphasizes the advantages of the use of recombinant proteins for serological testing. Moreover, insight into the possible future direction of these methods is also provided.

## Serodiagnosis of Toxoplasmosis

As a direct demonstration of the *T. gondii* parasite is often difficult, several serodiagnostic methods have been developed. These methods, which detect different antibodies (Montoya, 2002; Sudan et al., 2013) or antigens (Desmonts et al., 1981) have been used to achieve reliable diagnosis. In most epidemiological studies of toxoplasmosis, serological tests have been mainly preferred (Montoya, 2002; Robert-Gangneux and Dardé, 2012) and appear to be the primary approach in satisfactorily evaluating disease investigations (Rorman et al., 2006). The generation of each isotype antibodies is directly related to the humoral immune response after the infection. Hence, determining whether or not the host has *Toxoplasma* infection can be achieved by monitoring these responses. Due to the non-specificity of clinical signs of toxoplasmosis, serological test results have been paired with clinical signs evaluation in diagnosing toxoplasmosis (Montoya, 2002; Lopes et al., 2007).

The levels of different types of antibodies, including IgM, IgG, IgA, and IgE, are measured by the tests, which increases and decreases during or after infection (Rorman et al., 2006; Dubey, 2008). IgM is serologically detected 1 week after infection, and hence, is considered as an early and sensitive diagnostic marker for acute toxoplasmosis. However, it may also be serologically present for several months or years (Liu et al., 2015). In an infected pregnancy, IgM antibodies in the maternal circulation can be detected even 18 months after infection and may confuse interpretation whether the detected antibody is from active or previous infection (Bortoletti Filho et al., 2013). If an antibody is from an earlier infection, usually, no consequences for the fetus occur. However, if the infection occurs during pregnancy, the clinician should decide on administering anti-parasitic treatment to avoid disease complications in the unborn child (Montoya, 2002; Lopes et al., 2007). Results interpretation based on IgM levels can, therefore, be sometimes tricky and insufficient (Liu et al., 2015).

IgG antibodies against *T. gondii* can be detected 1–2 weeks following infection. It peaks typically within 1–2 months and declines at various rates. As it can persist lifelong at residual titers, this antibody is an indicator of a previous infection. It has since been used as a standard diagnostic marker for chronic infection. However, this antibody still has difficulty in differentiating previous and recent infections. An auxiliary IgG based-test has been established to differentiate acute from chronic infection in an asymptomatic patient (Montoya, 2002; Lopes et al., 2007). Other tests based on IgE and IgA have been developed. These antibodies are produced during the first weeks of infection and disappear early (Robert-Gangneux and Dardé, 2012). Various serological procedures have already been established to determine recent and previous exposures: Sabin-Feldman dye test (SFDT) (Sabin and Feldman, 1948), agglutination tests (Dubey, 1997, 2008; Robert-Gangneux and Dardé, 2012; Liu et al., 2015), indirect fluorescent assay (IFA) (Rorman et al., 2006; Saraei et al., 2010), and enzyme-linked immunosorbent assays (ELISAs) (Voller et al., 1976; Döskaya et al., 2014; Liu et al., 2015), or a combination of these methods (Rorman et al., 2006; Dubey, 2008; Robert-Gangneux and Dardé, 2012).

## Sabin-Feldman Dye Test (SFDT)

The Sabin-Feldman dye test (SFDT) was developed more than seven decades ago (Sabin and Feldman, 1948) for the investigation of *T. gondii* infection in the laboratory (Rorman et al., 2006). SFDT has high sensitivity and specificity and is still considered as the “gold standard” (Reiter-Owona et al., 1999). It utilizes complementation of live tachyzoite incubation with patient serum. If the serum has specific antibodies against *T. gondii*, the parasites will be subsequently coated and lyzed by the complement system, and staining with dye methylene blue will not happen. The number of stained (live) and unstained (dead) tachyzoites are counted to determine the end-point titer (Reiter-Owona et al., 1999; Rorman et al., 2006; Udonsom et al., 2010). While SFDT can detect both IgM and IgG, the antibody titers cannot accurately differentiate between acute or chronic infection. Moreover, SFDT entails using live parasites, which is a biohazard, thereby limiting its application to only a few laboratories (Reiter-Owona et al., 1999; Udonsom et al., 2010).

## Agglutination Tests

Agglutination tests require particulate antigens that can bind with antibodies. Multivalent antibodies (called agglutinins) form large clumps or aggregates with suspended particulate antigens when present, which can be visually seen without magnification. These tests are used to determine concentrations of specific antibodies. In toxoplasmosis diagnosis in humans and animals, different agglutination tests, including direct agglutination test (DAT), modified agglutination test (MAT), indirect hemagglutination test (IHAT), and latex agglutination test (LAT), have been used (Dubey, 1997, 2008; Robert-Gangneux and Dardé, 2012; Liu et al., 2015). DAT was developed in 1965 and has since been very useful in detecting anti-*T. gondii* antibodies in humans and animals (Dubey, 2008). It is only used for the detection of IgG antibodies. It is very simple as it does not require a secondary antibody and specialized equipment. In DAT, diluted patient sera are added to microtiter plates that are coated with formalinized *Toxoplasma* tachyzoites. Subsequent agglutination happens if anti-*Toxoplasma* antibodies are present in the sera. If the sample is negative, precipitated tachyzoites will be found at the bottom of the wells (Desmonts and Remington, 1980). While DAT is considered very sensitive and economical, it requires a large antigen amount. Moreover, the presence of IgM antibodies in the sera causes non-specific agglutination (Dubey, 2008). The MAT is an adaptation of the DAT with some adjustments involving the preparation of the antigen and incubation period of the test plates (Dubey, 1997; Al-Adhami et al., 2016).

IHAT utilizes red blood cells (RBCs) that are sensitized with *T. gondii* soluble antigen. The sensitized cells will subsequently agglutinate if the sera contain anti-*T. gondii* antibodies. IHAT is also considered very simple and inexpensive (Liu et al., 2015). On the other hand, LAT utilizes covalently bonded tachyzoite particles that are coated to latex beads. A visible agglutination reaction is observed when the sera contain specific IgG antibody (Mazumder et al., 1988). The sensitivity and specificity of LAT in humans and animals range from low to high. Same with other agglutination tests, non-specific IgM agglutinations can also happen in LAT which can generate false-positive results (Ohshima et al., 1981; Mazumder et al., 1988; Oncel et al., 2005; Robert-Gangneux and Dardé, 2012). LAT has been used often as a screening test for epidemiological studies before other serological tests are utilized for further examination (Holliman et al., 1990).

## Indirect Fluorescent Assay (IFA)

IFA is an alternative simple and safe diagnostic method that does not use live tachyzoites (Rorman et al., 2006; Saraei et al., 2010). This assay is based on the specific antigen-antibody interaction from diluted serum specimens with killed *Toxoplasma* tachyzoites. The interaction will then be detected by the addition of fluorescent-labeled anti-human IgG or IgM antibodies under a fluorescence microscope (Pappas et al., 1986). Among the limitations of IFA include the individual differences in result reading and the chances of false-positive results in case the sera contain rheumatoid factors or antinuclear antibodies (Rorman et al., 2006). Nonetheless, high levels of *T. gondii*-specific IgG in some recently acquired toxoplasmosis patients may interfere with the IgM antibodies and cause false-negative results (Remington et al., 1985).

## Enzyme-Linked Immunosorbent Assay (ELISA)

Even after four decades since it was established in toxoplasmosis diagnosis (Voller et al., 1976), ELISA is still considered one of the most common techniques with high sensitivity and specificity in the quantitative detection of antibodies and all antigenically active molecules (Döskaya et al., 2014; Liu et al., 2015). The ELISA system typically consists of a solid phase antigen or antibody, enzyme-labeled antigen or antibody, and a substrate for the enzyme reaction, which can be modified to test both antibodies and antigens (Liu et al., 2015). There are different kinds of ELISA developed to detect *T. gondii* antibodies or antigens, namely indirect ELISA, sandwich ELISA, and dot-ELISA. The indirect ELISA involves coating a microtiter plate with antigens and the application of sera, which contains antibodies. The presence of anti-*Toxoplasma* antibodies leads to consequent binding with the coated antigen and is detected by using an anti-human enzyme-conjugate (secondary antibody). The subsequent washing steps will remove any unbound reagents, and when the substrate is finally added, color reaction develops. This type of ELISA is mostly used to detect anti-*T. gondii* IgG, IgM, and IgA antibodies rather than antigens (Tomasi et al., 1986). The conventional indirect ELISAs using *T. gondii* tachyzoite lysate antigen (TLA) as coating antigen revealed a high degree of agreement with SFDT, MAT or IFAT detecting IgG or IgM antibodies in humans and animals (Filice et al., 1983; Tomasi et al., 1986; Obwaller et al., 1995). In the sandwich ELISA, capture antibodies are coated onto a microtiter plate, and a serum sample containing *T. gondii* antigens is added. After incubation and washing, the capture antibody-antigen reaction is also detected by the addition of enzyme-conjugated secondary antibody. Following subsequent washings, the substrate is added for a color reaction to develop. The sandwich ELISA with TLA is more sensitive and more specific to detect human IgM antibodies than IFAT (Tomasi et al., 1986). The dot-ELISA is a modified ELISA where the antigen-antibody reaction is done on nitrocellulose instead of the microtiter plate. This test is sensitive to detect *T. gondii* antigens and antibodies (Pappas et al., 1986; Jafar Pour Azami et al., 2011) and does not require any special equipment, thus easier to perform than standard ELISAs (Pappas et al., 1986; Youssef et al., 1992). The quantity of antibodies detected in the sera using ELISA is shown to be positively correlated with the intensity of the color reaction. Results interpretation usually is dependent on the qualitative assessment of color change spectrophotometrically. Deciding samples to be positive or negative is achieved by correlating the optical density of the serum with the control after establishing a threshold value (Seefeldt et al., 1989).

The ELISA is primarily utilized for routine screening of *T. gondii* infections because it is highly sensitive (allowing quantitative and semi-quantitative antibody measurements), easily adopted, and inexpensive (Shaapan et al., 2008). It can be simply used to test large populations in a short period of time (Sudan et al., 2013), with the capability to detect both IgG and IgM (Seefeldt et al., 1989). This method is also primarily used to evaluate the efficacy of different recombinant proteins as antigens for serodiagnosis. However, standardization of used antigens in ELISA has been challenging (Shaapan et al., 2008). In cases of a weak positive reaction, a photometer is required to differentiate it from a negative reaction, thereby increasing the cost (Seefeldt et al., 1989). False-positive results can also happen in IgM-based ELISA (Fuccillo et al., 1986; Liesenfeld et al., 1997), possibly due to rheumatoid factors in the serum, while IgG-based ELISA can result in false-negative results possibly due to specific IgG competitive inhibition (Fuccillo et al., 1986). The low-level IgG detection is a problem, i.e. IgG results from the “gray zone” in ELISA. According to Robert-Gangneux and Dardé (2012), these low titers must be confirmed by using a dye test or a sensitive Western blot (WB) assay.

## Immunochromatographic Tests (ICT)

ICT is a rapid lateral flow test intended to detect the presence or absence of the target analyte. The principle of ICT is based on a dye-labeled antibody or colloidal gold-labeled antigen that is specific for the target analyte in the liquid sample, which is present on the lower end of the nitrocellulose strip or in the plastic well along with the strip. The sample is placed at the designated pad on the nitrocellulose membrane, which will slowly infiltrate the conjugated pad through capillary action, and subsequent antibody-antigen complexes will demonstrate color reaction (Wang et al., 2011). It is simple because specialized and costly equipment may not be needed, although several laboratory-based applications and reading equipment may exist. It is believed to be a low-cost test which facilitates the rapid identification of analytes at the point of care (Weiss, 1999; Zhang et al., 2009; Thobhani et al., 2010; Goni et al., 2012; Yetisen et al., 2013). Its ease of application and rapidity of test results with no special equipment required makes the ICT suitable for field application. In toxoplasmosis, this technology has been used to diagnose human (Lévêque et al., 2019; Taha et al., 2019; Wassef and Abdel-Malek, 2019) and animal cases (Khan and Noordin, 2019). ICT has been shown to have a high agreement with ELISA in terms of sensitivity and specificity (Terkawi et al., 2013; Ybañez and Nishikawa, 2020; Ybañez, Kyan and Nishikawa, 2020). ICT based on GRA7 (Terkawi et al., 2013; Ybañez and Nishikawa, 2020; Ybañez, Kyan and Nishikawa, 2020) and SAG2 (Huang et al., 2004) show high consistency with results obtained from LAT and ELISA.

## Western Blotting

The western blot (sometimes referred to as immunoblot) aids conventional serological tests and shows the reaction of sera with *T. gondii* antigen on a membrane transferred from a polyacrylamide gel, and the resulting banding patterns that are matched with known molecular weight. An immunoblot test can have varying reliability of its specificity and sensitivity depending on the type of sample used (Villard et al., 2003; Stroehle et al., 2005). Western blot is also complementary for the early postnatal diagnosis of congenital toxoplasmosis (Robert-Gangneux et al., 1999), diagnosis of human patients (Gay et al., 2019) and characterization and evaluation of *T. gondii* proteins (Appiah-Kwarteng et al., 2019; Liu et al., 2019).

## Production of Specific and Standard Antigens for Serological Diagnosis of Toxoplasmosis

While *T. gondii* diagnosis based on serology are generally satisfactory, it has been challenged with producing specific and standard antigens that are usually crudely prepared through mouse passages or cell culture systems in commercial tests (Titilincu et al., 2009; Cóceres et al., 2010; Dai et al., 2012; Sudan et al., 2013; Sun et al., 2013). It has been shown that the different processes of producing and purifying native antigens may lead to contamination with non-parasitic materials (Holec-Gąsior, 2013). These processes also utilize live pathogens that require extra care because of biological hazards (Sonaimuthu et al., 2014) and are, therefore, difficult to standardize (Holec-Gąsior, 2013). With the limitations posed by the native antigens and the need to improve serodiagnostic tests, recombinant antigens have been considered as an alternative diagnostic marker to replace the native antigens (Cai et al., 2015).

## Recombinant Antigens

There are already several studies that have documented that recombinant antigens improve the serological diagnosis of toxoplasmosis (Harning et al., 1996; Martin et al., 1998; Jacobs et al., 1999; Aubert et al., 2000; Lecordier et al., 2000; Beghetto et al., 2003, 2006; Pietkiewicz et al., 2004; Buffolano et al., 2005; Hiszczyńska-Sawicka et al., 2005; Pfrepper et al., 2005; Holec et al., 2007; Lau and Fong, 2008; Holec-Gąsior et al., 2009; Wu et al., 2009; Holec-Gąsior and Kur, 2010; Sonaimuthu et al., 2014). However, the critical goal is not only to enhance *T. gondii* diagnosis with the use of recombinant antigens but also to improve ways to discriminate different stages of toxoplasmosis. Primary infection during pregnancy predisposes the child to serious medical problems (Petersen, 2007). The accurate diagnosis of the acute stage of the disease in pregnant women ensures early care for the mother and the fetus to prevent serious medical problems; thus, the differentiation between acute and chronic phases of *T. gondii* infection is vital (Ciardelli et al., 2008; Márquez-Contreras, 2018).

Through the years, several genes encoding *T. gondii* proteins have been cloned and expressed using various expression systems to produce recombinant antigens. The antigens that have been widely utilized for the improvement of *T. gondii* serodiagnosis include the surface antigens SAG1 (P30) (Burg et al., 1988; Chen et al., 2001; Hiszczyńska-Sawicka et al., 2003; Kotresha et al., 2012), SAG2 (previously P22) (Prince et al., 1990; Parmley et al., 1992; Hiszczyńska-Sawicka et al., 2005), SAG3 (previously P43) (Khanaliha et al., 2012); the rhoptries ROP1 (previously P66) (Holec-Gąsior et al., 2009) and ROP2 (previously P54) (Saavedra et al., 1991; van Gelder et al., 1993; Martin et al., 1998); the dense granule antigens GRA1 (previously P24) (Cesbron-Delauw et al., 1989; Hiszczyńska-Sawicka et al., 2003), GRA2 (previously P28) (Prince et al., 1989; Murray et al., 1993; Holec-Gąsior et al., 2009; Lau et al., 2012; Ching et al., 2013), GRA4 (Mévélec et al., 1992; Lau et al., 2010), GRA5 (Holec-Gąsior and Kur, 2010), GRA6 (previously P32) (Lecordier et al., 1995; Redlich and Müller, 1998; Hiszczyńska-Sawicka et al., 2005), and GRA7 (previously P29) (Bonhomme et al., 1998; Fischer et al., 1998; Jacobs et al., 1998; Hiszczyńska-Sawicka et al., 2003; Selseleh et al., 2012); GRA8 (previously P35) (Hiszczyńska-Sawicka et al., 2005), and GRA9 (previously B10/P41) (Nockemann et al., 1998), the matrix antigen MAG1 (Holec et al., 2007), and the micronene protein MIC1 (Holec et al., 2008).

The use of specific molecular markers is another option adapted in *T. gondii* serodiagnosis. These proteins are distinctive of the parasite's tachyzoite or bradyzoite stage that could recognize specific antibodies from acute or chronic human infections. Recently, many papers have reported positive results on the utility of specific recombinant proteins that identify the phase of infection during the testing of human sera. Table 1 details the application of *T. gondii* recombinant antigens in diagnostic studies, and their potential to recognize the clinical phases of the disease. Collectively, these results suggest that adequately selected recombinant antigens can be employed to investigate acute or chronic toxoplasmosis. In *T. gondii*, dense granule (GRA) proteins are vastly secreted into the parasitophorous vacuole (PV) shortly following host invasion. These proteins are the major components of *T. gondii* excretory-secretory antigens (ESA) expressed by both the tachyzoites and encysted bradyzoites and circulate in the bloodstream during the first few hours after infection (Hughes and van Knapen, 1982; Cesbron-Delauw, 1994) It has been shown that ESAs are highly immunogenic (Darcy et al., 1988; Prigione et al., 2000), and can stimulate an antibody-dependent or cell-mediated immunity (Zenner et al., 1999). GRA antigens such as the GRA1, 2, 3, 4, 5, 6, 7, and 8 have been evaluated for its potential as molecular markers for the detection of antibodies against *T. gondii*, and all showed high sensitivities for the detection of anti-*Toxoplasma* antibodies (Table 1). However, GRA2 (Holec-Gąsior et al., 2009), GRA6 (Redlich and Müller, 1998; Hiszczyńska-Sawicka et al., 2005; Golkar et al., 2008), GRA7 (Pietkiewicz et al., 2004; Kotresha et al., 2012), and GRA8 (Li et al., 2000b; Suzuki et al., 2000; Lu et al., 2006) proved to be valuable markers for the diagnosis of acute toxoplasmosis, while GRA5 showed high sensitivity to detect IgG antibodies from individuals with chronic toxoplasmosis (Holec-Gąsior and Kur, 2010). The surface antigens (SAG) of *T. gondii* are abundant on the surface of both extracellular and intracellular tachyzoites (Burg et al., 1988). The SAG1 is one of the most immunogenic *T. gondii* antigens widely used for its diagnostic ability (Pietkiewicz et al., 2004; Jalallou et al., 2010; Bel-Ochi et al., 2013). It is reported to be stage-specific, being detected only in the tachyzoite stage, and not in the sporozoite and bradyzoite stages (Burg et al., 1988; Windeck and Gross, 1996). However, previous serodiagnosis studies revealed that SAG1 is a useful antigen for the diagnosis of chronic toxoplasmosis (Pietkiewicz et al., 2004; Selseleh et al., 2012). Contrastingly, a study using another surface antigen, SAG2, revealed that IgG from patients with acute phase of toxoplasmosis reacted much more with SAG2A antigens than sera from patients with a chronic phase, confirming its potential as a marker for diagnosis of human acute toxoplasmosis (Béla et al., 2008). The rhoptry proteins (ROP) are another secretory antigens of *T. gondii* that are reported to be also involved in the formation of the PV and the clustering with host cell organelles (Sam-Yellowe, 1996). Among the ROP antigens evaluated for its serodiagnostic utility, the ROP1 (Holec-Gąsior et al., 2009, 2010), ROP8 (Sonaimuthu et al., 2014), and ROP18 (Grzybowski et al., 2015) showed higher antibody reactions, with ROP1 and ROP18 being able to detect antibodies from individuals with acute toxoplasmosis.

**Table 1:** Updated list of *T. gondii* recombinant antigens used as molecular markers for the serodiagnosis of human toxoplasmosis.

**Antigen**	**Expression system^**a**^**	**Diagnostic test**	**No. and details of tested sera**	**Results**	**References**
GRA1 (Formerly P24)	Recombinant protein with His tag domain	IgG	117 total; 45 acute, 72 chronic	Sensitivity, 83.3% (acute-phase), 77.8% (chronic sera)	Pietkiewicz et al., 2004
GRA2 (Formerly P28)	Recombinant protein with TRX and His tag domains	IgG ELISA	59 total pregnant women in France (24 acute, 35 chronic) 46 total pregnant women in Iran (18 acute, 28 chronic)	Sensitivity, 95.8% (acute-phase), 65.7% (chronic sera) Sensitivity, 100% acute-phase sera, 71.4% (chronic sera)	Golkar et al., 2007
	Recombinant protein with His tag domain	IgG ELISA	127 total; 37 acute, 90 chronic	Sensitivity, 100% (acute-phase), 22.5% (chronic sera)	Holec-Gąsior et al., 2009
GRA3	Recombinant protein with GST domain	IgG ELISA	130 total; 100 adults, 30 infants from infected mothers	Sensitivity, 82%; specificity, 100%	Beghetto et al., 2006
GRA4 (Formerly P41)	Recombinant protein with His tag domain	IgG ELISA	36 total; 12 acute,127 total; 37 22 chronic	Sensitivity, 58.3% (acute-phase), 18.2% (chronic sera)	Nigro et al., 2003
GRA5	Recombinant protein with His tag domain	IgG ELISA	189 total; 27 acute, 18 post-acute, 144 chronic	Sensitivity, 70.9% overall; 63% (acute-phase), 50% for post-acute, 75% (chronic sera)	Holec-Gąsior and Kur, 2010
GRA6 (Formerly P32)	Recombinant protein with GST domain	IgG ELISA	298 total; 61 acute, 132 chronic, 100 no serological evidence, 5 with persistent IgM antibodies	Sensitivity, 86% (acute-phase); Sensitivity 89%; specificity, 99.6% between presence and absence of acute infection	Redlich and Müller, 1998
	Recombinant protein with His tag domain	IgG ELISA	90 total, 33 acute,127 total; 37 57 chronic	Sensitivity, 93.9% (acute-phase), 63.1% (chronic sera)	Hiszczyńska-Sawicka et al., 2005
	Recombinant protein with His tag domain	IgG ELISA	58 total pregnant; 24 acute (seroconversion during 4 mos prior to sampling), 34 chronic	*For cutoff value d of mean + 2 SD Sensitivity, 95.8% (acute-phase), 44.1% (chronic sera) *For cutoff value d of mean + 3 SD Sensitivity was 87.5% (acute-phase), 5.9% for chronic sera	Golkar et al., 2008
	Recombinant protein with His tag domain	IgG avidity	37 total pregnant women in France (20 acute, 17 chronic) 69 total pregnant women in Iran (35 acute, 34 chronic)	100% of chronic sera displayed high-avidity IgG antibodies against sera collected more than 4 mos after infection	Elyasi et al., 2010
GRA7 (Formerly P29)	Recombinant protein with His tag domain	IgG ELISA	180 total, 95 acute, 85 chronic	Sensitivity, 94% (acute-phase), 79% (chronic sera); specificity, 98%	Jacobs et al., 1999
	Recombinant protein with CKS tag	IgG ELISA	247 total, 89 acute, 105 chronic, 53 recent seroconversion	Sensitivity, 83.4%	Aubert et al., 2000
	Recombinant protein with His tag domain	IgG ELISA	36 total, 12 acute, 22 chronic	Sensitivity, 75% (acute-phase), 36.3% (chronic sera)	Nigro et al., 2003
	Recombinant protein with His tag domain	IgG ELISA	117 total; 45 acute, 72 chronic	Sensitivity, 95.9% (acute-phase), 68.9% (chronic sera)	Pietkiewicz et al., 2004
	Recombinant protein with GST domain	IgG ELISA	100 adults; 30 infants from infected mothers	Sensitivity, 88%; specificity, 100%	Beghetto et al., 2006
	Recombinant protein with His tag domain	IgG immunoblot	40 total; 20 acute, 20 chronic	Sensitivity, 100% (acute-phase); 40% for chronic sera	Kotresha et al., 2012
GRA8 (Formerly P35)	Recombinant protein with CKS domain	IgG ELISA	247 total, 89 acute, 105 chronic, 53 recent seroconversion	Sensitivity, 70%	Aubert et al., 2000
		IgM ELISA	142 total, 105 chronic, 53 recent seroconversion	Sensitivity, 54.9%	
	Recombinant protein with GST domain	IgM ELISA	53 total; 20 acute, 33 chronic	Sensitivity, 90% (acute-phase); no chronic sera were positive	Suzuki et al., 2000
	Recombinant protein with CKS domain	IgG ELISA	80 total pregnant women; 41 acute, 50 chronic	Sensitivity, 85.3% (acute-phase), 8% (chronic sera)	Li et al., 2000b
	Recombinant protein with His tag domain	IgG ELISA	90 total; 33 acute, 57 chronic	Sensitivity, 86.7% (acute-phase), 54.5% (chronic sera)	Hiszczyńska-Sawicka et al., 2005
	Recombinant protein with GST domain	IgM ELISA	100 total; 25 acute, 25 recently seroconverted, 25 persistent IgM-positive, 25 chronic	Sensitivity, 100% (acute-phase), 88% (recently seroconverted), 16% (persistent IgM-positive), 4% (chronic sera)	Lu et al., 2006
M2AP	Recombinant protein with GST domain	IgG ELISA	130 total; 100 adults, 30 infants from infected mothers	Sensitivity, 78%; specificity, 100%	Beghetto et al., 2006
MAG1	Recombinant protein with His tag domain	IgG ELISA	117 total; 37 acute, 80 chronic	Sensitivity, 97.3% (acute-phase), 7.5% (chronic sera)	Holec et al., 2007
MIC2	Recombinant protein with GST domain	IgG ELISA	130 total; 100 adults, 30 infants from infected mothers	Sensitivity, 92%; specificity, 100%	Beghetto et al., 2006
MIC3	Recombinant protein with GST domain	IgG avidity	121 total; 80 pregnant women with seroconversion	Low avidity of IgG antibodies in sera collected within 2 mo after infection	Beghetto et al., 2003
	Recombinant protein with GST domain	IgG ELISA	130 total; 100 adults, 30 infants from infected mothers	Sensitivity, 90%; specificity, 100%	Beghetto et al., 2006
P68	Recombinant protein with CKS domain	IgG ELISA	247 total; 89 acute, 105 chronic, 53 recent seroconversion	Sensitivity, 69.2%	Aubert et al., 2000
		IgM ELISA	142 total; 105 chronic, 53 recent seroconversion	Sensitivity, 18.3%	
ROP1 (Formerly P66)	Recombinant protein with CKS domain	IgG ELISA	247 total; 89 acute, 105 chronic, 53 recent seroconversion	Sensitivity, 59.1%	Aubert et al., 2000
	Recombinant protein with CKS domain	IgM ELISA	142 total; 105 chronic, 53 recent seroconversion	Sensitivity, 58.5%	
	Recombinant protein with His tag domain	IgG ELISA	127 total; 37 acute, 90 chronic	Sensitivity, 94.6% (acute-phase), 15.5% (chronic sera)	Holec-Gąsior et al., 2009
	Recombinant protein with CKS domain	IgG avidity	172 total for routine screening	Sensitivity, 85% (acute-phase), 25% (chronic sera)	Holec-Gąsior et al., 2010
ROP2 (Formerly P54)	Recombinant protein with CKS domain	IgG ELISA	247 total; 89 acute, 105 chronic, 53 recent seroconversion	Sensitivity, 58.3%	Aubert et al., 2000
		IgM ELISA	142 total; 105 chronic, 53 recent seroconversion	Sensitivity, 12.7%	
	Recombinant protein with His tag domain	IgG ELISA	36 total; 12 acute, 22 chronic	Sensitivity, 83.3% (acute-phase), 54.5% (chronic sera)	Nigro et al., 2003
ROP5	Recombinant protein with His tag domain	IgG ELISA	146 total; 34 acute, 86 chronic, 26 seronegative	Sensitivity, 23%; Specificity, 100%	Grzybowski et al., 2015
		IgM ELISA		Sensitivity, 26%; Specificity, 91%	
ROP8	Recombinant protein with His tag domain	IgG Western Blot	105 total; 25 early acute, 30 acute, 50 chronic	Sensitivity, 90% overall; 90% (early acute-phase), 92% (acute sera), 82% (chronic sera); specificity, 94%	Sonaimuthu et al., 2014
ROP18	Recombinant protein with His tag domain	IgG ELISA	146 total; 34 acute, 86 chronic, 26 seronegative	Sensitivity, 38%; specificity, 93%,	Grzybowski et al., 2015
		IgM ELISA		Sensitivity, 68%, specificity, 76%,	
SAG1 (Formerly P30)	Recombinant protein with CKS domain	IgG ELISA	247 total, 89 acute, 105 chronic, 53 recent seroconversion	Sensitivity, 74.1%	Aubert et al., 2000
		IgM ELISA	142 total; 105 chronic, 53 recent seroconversion	Sensitivity, 10.6%	
	Recombinant protein with His tag domain	IgG ELISA	117 total; 45 acute, 72 chronic	Sensitivity, 98.6% (acute-phase), 100% (chronic sera)	Pietkiewicz et al., 2004
	Recombinant protein with GST domain	IgG ELISA	130 total; 100 adults, 30 infants from infected mothers	Sensitivity, 82%; specificity, 100%	Beghetto et al., 2006
	Recombinant protein with His tag domain	IgG ELISA	173 total patients; 153 (with suspected *T. gondii* infection), 20 (with other diseases)	Sensitivity, 88.4%; specificity, 88%	Jalallou et al., 2010
	Recombinant protein with His tag domain	IgG ELISA	204 total, 74 IgG positive, 70 IgM positive; 60 no serological evidence	Sensitivity, 93%, specificity, 95%	Selseleh et al., 2012
		IgM ELISA		Sensitivity, 87%, specificity, 95%	
	Recombinant protein with His tag domain	IgG ELISA	91 total pregnant; 49 seropositive, 42 Seronegative	Sensitivity, 100%, specificity, 100%	Bel-Ochi et al., 2013
SAG2 (Formerly P22)	Recombinant protein with His tag domain	IgG ELISA	90 total; 33 acute, 57 chronic	Sensitivity,95.6% overall; 93.9% (acute-phase), 96.5% (chronic sera)	Hiszczyńska-Sawicka et al., 2005
	Recombinant protein with His tag domain in yeast *Pichia pastoris*	IgG /IgM ELISA	60 total; 20 early acute, 20 acute, 20 chronic	Sensitivity, 80% (early acute-phase), 95% (acute sera), 100% (chronic sera); specificity, 100%	Lau and Fong, 2008
SAG2 A	Recombinant protein with His tag domain	IgG ELISA IgG avidity	60 total; 30 acute, 30 chronic	Sensitivity, 95%, overall; 90% (acute-phase), 67% (chronic sera); specificity, 100%	Béla et al., 2008

a *All recombinant proteins were produced in E. coli unless specified*.

The use of specific recombinant antigens appears to be promising and sensitive enough in differentiating acute vs. chronic infection (Ferrandiz et al., 2004; Ching et al., 2014). Despite no clear definition of either chronic or acute infection, detection of IgM antibodies is usually employed for the diagnosis of acute infection in immunocompetent patients (Remington et al., 2011). IgM antibodies appear earlier in infection but decline more rapidly than IgG antibodies, enabling its detection to determine recent or previously acquired infection. Nevertheless, IgM might remain for a long time post-infection, even detectable 2 years after infection (Bobić et al., 1991). Therefore, the results may not be correct to prove recent infection, unless the serum is tested further using another method such as the IgG avidity. The IgG avidity assay, based on functional affinity of IgG antibodies (Hedman et al., 1993), may be used to detect recently acquired (acute) or latent (chronic) infection that has been in the body for a longer period (Hedman et al., 1993; Rahbari et al., 2012) The avidity of IgG is low in acute phase and high in chronic phase of toxoplasmosis (Iqbal and Khalid, 2007); high IgG avidity excludes infection three to five months prior, while a low avidity indicates recent infection (Flori et al., 2008).

In 2013, the diagnostic performances of four commercially available *Toxoplasma* IgG avidity tests, ARCHITECT Toxo IgG Avidity (Abbott, Wiesbaden, Germany), Vidas Toxo IgG Avidity (bioMérieux, Marcy l'Étoile, France), Platelia Toxo IgG Avidity (Bio-Rad, Marnes la Coquette, France), and Liaison Toxo IgG Avidity II (DiaSorin, Saluggia, Italy), were assessed in immunocompetent and immunocompromised patients with acute and latent toxoplasmosis in France (Villard et al., 2013). These fully automated assays are the most commonly used in French biology laboratories and reference laboratories abroad. They were developed based on the exclusion of acute infection. Among the four tests, the Vidas Toxo IgG Avidity showed the best performance for the diagnosis of latent toxoplasmosis. The ARCHITECT assay, which utilizes tachyzoite-specific surface antigen P30 (SAG1) and P35 (GRA8) recombinant antigens, performed best in the detection of latent infection in the presence of persistent IgM. The test detects low-avidity IgG by blocking high-avidity IgG in the sample with a soluble recombinant antigen (Curdt et al., 2009). Sickinger et al. (2008) also confirmed that the ARCHITECT Toxo IgG and IgG avidity panel can be used to rule out acute T. gondii infection in pregnant women as it was able to detect 100% (124/124) of acute-phase sera (four months after infection) as low avidity, compared to the 98.9% detected by the Vidas Toxo IgG avidity assay. Based on these findings, new prospectives for *T. gondii* serodiagnosis can be offered by the application of recombinant antigens in an IgG avidity assay. Previously, proteins P16, P32, P38, P40, P43, P54, P60, P66, and P97 were selected as valuable antigens in an avidity assay to potentially discriminate between phases of toxoplasmosis (Marcolino et al., 2000). Beghetto et al. (2003) showed that MIC3 is an excellent molecular marker that distinguishes infection based on avidity results between sera from patients infected with *T. gondii* within or more than 2 months after infection (Table 1). An avidity test was constructed by applying ROP1, MAG1, SAG1, GRA7, and GRA8 antigens onto the recom-Line *Toxoplasma* IgG strip test (Mikrogen GmbH, Nueried, Germany). Results revealed that IgG antibodies against antigens recognized early (i.e., GRA7, GRA8, and ROP1) matured significantly earlier than those IgGs directed against antigens that were recognized later (i.e., SAG1 and MAG1) (Pfrepper et al., 2005). Another IgG avidity test that applied recombinant ROP1 antigen detected specific low-avidity antibodies in most of the sera from individuals with acute toxoplasmosis, high-avidity antibodies were detected in sera from patients with chronic infection (Holec-Gąsior et al., 2010). Meanwhile, a GRA6 avidity testing (Table 1) among pregnant women may be useful to rule out recent infections occurring 4 months prior (Elyasi et al., 2010). Moreover, a study utilizing a mixture of recombinant proteins, including GRA7, SAG1, and GRA1, for IgG avidity testing reported that IgG avidity maturation against this mixture is different from that received against TLAs. This finding corroborates with previous reports stating that the development of IgG avidity maturation varies depending on the stimulating antigen and antibodies mature at different rates than the *Toxoplasma* native antigens (Marcolino et al., 2000; Pfrepper et al., 2005; Pietkiewicz et al., 2007). All these findings highlight the enormous potential of recombinant antigens to replace TLAs in IgG avidity assays and enhance the current methods for serodiagnosis, especially for acute *T. gondii* infections.

## Combination of Recombinant Proteins

Aside from ease of standardization, another advantage of using recombinant proteins over whole parasite lysates is that more than one antigen can be applied at the same time, like in ELISA. In 1992, the diagnostic value of combining two recombinant *T. gondii* proteins for the detection of *T. gondii* specific IgM was evaluated for the first time (Johnson et al., 1992). An ELISA based on the combination of H4/GST and H11/GST revealed higher sensitivity (81.3%) for the detection of IgM ELISA as compared to when H4/GST and H11/GST were tested separately (54% and 61%, respectively) (Tenter and Johnson, 1991). Jacobs et al. (1999) evaluated the performance of recombinant GRA7 and Tg34AR (ROP2 C-terminal fragment) for the detection of IgG-specific antibodies. When used separately, the sensitivity of the ELISA was 81 and 88%, respectively, but the mixture of the two proteins improved the sensitivity to 96%. Similarly, Lecordier et al. (2000) discovered that GRA1 may complement GRA6-Nt to attain an overall IgG ELISA sensitivity of 98%. Low sensitivities were obtained when GRA1 and GRA6-Nt were applied individually (68 and 96%, respectively).

Table 2 shows different combinations of recombinant proteins that were recommended for the detection of IgM and IgG antibodies against *T. gondii*. A cocktail of GRA7, GRA8, and ROP1 recombinant proteins was reported beneficial to detect IgM antibodies in human sera (Aubert et al., 2000). Furthermore, other antigen mixtures such as MAG1 or GRA2 or ROP1 supplemented with SAG1 and GRA5 (Holec-Gąsior and Kur, 2010); MIC1ex2, MAG1, and MIC3, or P35 (GRA8), SAG2, and GRA6, (Holec et al., 2008); SAG1, GRA1, and GRA7 (Pietkiewicz et al., 2004); P22 (SAG2), P25 (H4), P29 (GRA7), and P35 (GRA8) (Li et al., 2000a); and GRA7, SAG1, and GRA8 (Aubert et al., 2000) were documented to be sufficient for the detection of IgG antibodies against *T. gondii*. All these data presented in the previous studies mentioned above have validated the potential of using two or more complementary recombinant antigens to improve the sensitivity of immunoassays comparable to that obtained with using crude antigens. It is noteworthy that the combinations of recombinant antigens mentioned above include any of the GRA5, GRA7, GRA8, SAG2, and H4 proteins that have been reported to be valuable in differentiating a recently acquired infection from one acquired in the past (Li et al., 2000a; Holec-Gąsior and Kur, 2010). The antigen concoctions presented above were established to be antigenic, with sensitivity for specific IgG or IgM detection comparable to native antigens of *T. gondii*. However, to optimize the detection of antibodies from different stages of the toxoplasmosis, the assay necessitates proportion of highly reactive antigens, such as SAG1 (Jalallou et al., 2010; Bel-Ochi et al., 2013), and specific molecular markers for acute-stage toxoplasmosis, like the GRA2 (Holec-Gąsior et al., 2009), GRA6 (Hiszczyńska-Sawicka et al., 2005), GRA7 (Pietkiewicz et al., 2004), GRA8 (Lu et al., 2006), MAG1 (Holec et al., 2007), and ROP1 (Holec-Gąsior et al., 2009, 2010); and GRA5 (Holec-Gąsior and Kur, 2010) and SAG1 (Pietkiewicz et al., 2004) for chronic stage serodetection. Therefore, a well-defined component of antigen mixtures is vital in obtaining a preparation that is essential for any serodiagnostic application.

**Table 2:** List of combinations of *T. gondii* recombinant antigens used for the serodiagnosis of human toxoplasmosis.

**Antigen mixture**	**Expression system^**a**^**	**Diagnostic test**	**No. and details of tested sera**	**Results**	**References**
GRA7-Tg34AR	Recombinant proteins with His tag domain	IgG /IgM ELISA	180 total, 95 acute, 85 chronic	Sensitivity: 96% (chronic sera)	Jacobs et al., 1999
P29 (GRA7), P30 (SAG1), P35 (GRA8)	Recombinant proteins with CKS domain	IgG ELISA	247 total; 89 acute, 105 chronic, 53 recent seroconversion	Sensitivity: 93.1%; specificity, 95.7%	Aubert et al., 2000
P29 (GRA7), P35 (GRA8), P66 (ROP1)	Recombinant proteins with CKS domain	IgM ELISA	142 total; 89 acute, 53 recent seroconversion	Sensitivity: 74.6%; specificity, 95.7%	
P22 (SAG2), P25 (H4), P29 (GRA7), P35 (GRA8)	Recombinant proteins with MBP domain (P22, P25, P29) and GST domain (P35)	IgG ELISA	96 total pregnant women; 20 acute, 70 chronic	Sensitivity, 90% (acute-phase), 98.6% (chronic sera); specificity, 97% (acute- phase)	Li et al., 2000b
SAG1, GRA1, GRA7	Recombinant proteins with His tag domain	IgG ELISA	241 total, 117 acute, 124 chronic	Sensitivity, 100% acute-phase sera, 91.1%, chronic sera	Pietkiewicz et al., 2004
MICex2, MAG1, MIC3,	Recombinant proteins with His tag domain	IgG ELISA	72 chronic sera	Sensitivity, 88.9%; specificity, 100%	Holec et al., 2008
P35 (GRA8), SAG2, GRA6				Sensitivity, 94.4%; specificity, 100%	
MAG1, SAG1, GRA5	Recombinant proteins with His tag domain	IgG ELISA	189 total; 27 acute, 18 post-acute, 144 chronic	Sensitivity, 92.6%; specificity, 100%	Holec-Gąsior and Kur, 2010
GRA2, SAG1, GRA5				Sensitivity, 93.1%; specificity, 100%	
ROP1, SAG1, GRA5				Sensitivity, 94.2%; specificity, 100%	

a *All recombinant proteins were produced in E. coli unless specified*.

## Recombinant Multi-Epitope and Chimeric Antigens

In recent years, the use of multi-epitope or chimeric antigens has been recommended as an alternative approach to address the need for standardizing and increasing the sensitivity and specificity of serodiagnostic tests of toxoplasmosis. Moreover, the capability to discriminate previous from recently acquired infections can also be accomplished (Wang et al., 2014). A recombinant chimera contains different immunoreactive epitopes from several properly selected *T. gondii* antigens that are generally well-exposed on the protein surface. The epitope or antigenic determinant is a part of a protein with an ability to be recognized by T cell and B cell receptors or the antibody binding sites (Saha and Raghava, 2006). The use of diagnostic markers with a high density of antibody binding sites increases the chances of antibody detection in serum samples and thereby enhances the degree of specificity and sensitivity of the assay (Camussone et al., 2009; De Souza et al., 2013). Numerous bioinformatic tools have been handy in the prediction and identification of immunodominant epitopes. The epitopes of several *T. gondii* antigens can be predicted, and their antigenicity can be evaluated by employing software-based prediction techniques (Dai et al., 2012; Wang et al., 2014). Phage display of cDNA libraries (Beghetto et al., 2001), epitope mapping (Cardona et al., 2009; Reineke, 2009), and reactivity with monoclonal antibodies (Mévélec et al., 1998) are among the experimental approaches applied to identify epitopes.

Presently, only minimal studies have described the diagnostic usefulness of different chimeric proteins for the serodiagnosis of toxoplasmosis in human sera (Table 3). Beghetto et al. (2006) first explored the application of two chimeric antigens namely, GST-EC2 and GST-EC3, which contains antigenic regions of MIC2, MIC3, SAG1, GRA3, GRA7, and M2AP proteins. The study revealed that both chimeric antigens obtained an improved serodiagnosis of toxoplasmosis in adults with acquired infection and infants born to mothers with a primary *T. gondii* infection. Additionally, the performance of the IgG and IgM Rec-ELISAs based on the two chimeric antigens were comparable to those of the commercial assays. Moreover, the IgM Rec-ELISAs using the GST-EC2 and GST-EC3 chimeric antigens improved the capacity to diagnose congenital toxoplasmosis postnatally compared to standard assays. In 2011, the specificity of the recombinant chimeric SAG1/2 antigen to detect IgG and IgM in *T. gondii* infection was confirmed using Western blot. Results revealed that it was immunogenic enough to stimulate a humoral response and protection in a mouse model and might be considered a good vaccine candidate (Lau et al., 2011). Meanwhile, Holec-Gąsior et al. (2012a) designed a chimera containing antigenic regions of MIC1 and MAG1 proteins. A high sensitivity using MIC1-MAG1 chimeric protein (90.9%) was attained, which was almost as high as that for the TLA (91.8%), and higher than the sensitivities of the assays using the recombinant proteins individually or a mixture of both which ranged 60–75.5% only. Furthermore, another chimeric protein containing immunodominant regions from MIC1, MAG1, and SAG1 (Holec-Gąsior et al., 2012b) was developed by the same research group and generated better results than the chimeric antigen containing fragments only from MIC1 and MAG1 proteins (Table 3). The addition of a fragment of SAG1, one of the most immunogenic proteins of *T. gondii*, to the chimeric antigen increased the reactivity with specific IgG antibodies from patients with chronic toxoplasmosis. These suggest that a properly designed chimeric antigen containing numerous different immunogenic regions is better than a mixture of recombinant proteins and may be used instead of TLAs for optimal serodiagnosis human *T. gondii* infections. Using the software, SAG1, SAG2, SAG3, GRA5, GRA6, and P35 were analyzed to identify immunodominant epitopes for the serodiagnosis of *T. gondii* infection. Two potential epitopes with high predicted antigenicity and reactivity were selected for each antigen. *T. gondii*-positive human sera strongly recognized three recombinant epitopes (rEPs), cloned from SAG1 (rSAG1_EP2), SAG2 (rSAG2_EP1), and SAG3 (rSAG3_EP2) antigens. A recombinant multi-epitope fusion peptide (rMEP) consisting of these three epitopes was then developed and assessed using IgG and IgM ELISAs (Dai et al., 2012, 2013). The results revealed that the rMEP successfully discriminated sera of pregnant women with recent and past infections, and obtained similar serodiagnostic efficiency as the two commercially available ELISA kits. In 2015, a chimeric antigen made up of antigenic fragments of SAG2, GRA1, and ROP1 (large fragment, ROP1_L_) achieved 100% sensitivity and specificity when utilized in an IgG ELISA assay (Ferra et al., 2015). In the same year, a synthetic gene called USM.TOXO1 that encodes multi-immunodominant epitopes of SAG1, GRA2, and GRA7 was constructed by assembly PCR (Hajissa et al., 2015). Initial ELISA and Western blot analyses using 80 human serum samples showed 100% sensitivity and specificity. The efficacy of USM.TOXO1 was further validated by testing 157 human sera and obtained sensitivity and specificity of 85.43 and 81.25%, respectively (Hajissa et al., 2017). The latest addition to the pool of suitable chimeric proteins for the detection of specific anti-*T. gondii* antibodies are the recombinant tetravalent chimeric proteins containing fragments of SAG2, GRA1, ROP1, and AMA1 antigens (Ferra et al., 2019). In this study, four tetravalent recombinant chimeric proteins (SAG2-GRA1-ROP1-AMA1N, AMA1N-SAG2-GRA1-ROP1, AMA1C-SAG2-GRA1-ROP1, and AMA1-SAG2-GRA1-ROP1) acquired through genetic engineering were evaluated for their efficacy in detecting specific IgM and IgG antibodies from *T*. *gondii-*infected human sera. All chimeric proteins showed 100% sensitivity and specificity in the IgG ELISAs. Avidity assay results suggested the usefulness of the chimeric antigens for avidity assessment, with results comparable to commercial assays. Furthermore, the AMA1-SAG2-GRA1-ROP1 chimeric protein displayed great potential in distinguishing specific antibodies from the sera of individuals with acute and chronic *T. gondii* infections. These findings exhibit the potential application of these recombinant chimeric antigens as replacements for TLA in standardized commercial tests for toxoplasmosis serodiagnosis.

**Table 3:** Updated list of multi-epitope and chimeric *T. gondii* recombinant antigens used for the serodiagnosis of human toxoplasmosis.

**Antigen**	**Antigenic regions (AA)**	**Expression system^**a**^**	**Diagnostic test**	**No. and details of tested sera**	**Results**	**References**
GST-EC2	157–235 MIC2 234–307 MIC3 182–312 SAG1	Recombinant protein with GST domain	IgM ELISA IgG ELISA	170 total; 50 adults with acquired *T. gondii* infection, 20 infants from mothers with primary *T. gondii* infection; 100 adults with acquired infection	Sensitivity, 98% (adults), 70% (infants) Sensitivity and specificity, 100%	Beghetto et al., 2006
GST-EC3	36–134 GRA3 24–102 GRA7 37–263 M2AP		IgM ELISA IgG ELISA		Sensitivity, 84% (adults), 50% (infants) Sensitivity and specificity, 100%	
SAG1+SAG2	1-336 SAG1 1-186 SAG2	Recombinant protein with His tag domain in yeast *Pichia pastoris*	IgG/IgM Western blot	110 total; 20 early acute, 20 acute, 20 chronic	Sensitivity and specificity, 100%	Lau et al., 2011
MIC1+MAG1	25-182 MIC1 30-222 MAG1	Recombinant protein with His tag domain	IgG ELISA	110 total; 26 acute, 17 postacute, 67 chronic	Sensitivity, 90.9% overall; 100% (acute and postacute-phase), 85.1% (chronic sera); specificity, 100%	Holec-Gąsior et al., 2012a
MIC1+MAG1+SAG1	25-182 MIC1 30-222 MAG1 49-198 SAG1	Recombinant protein with His tag domain	IgG ELISA	162 total; 47 acute, 19 postacute, 96 chronic	Sensitivity, 98.1% overall; 100% (acute and postacute-phase), 96.9% (chronic sera); specificity, 100%	Holec-Gąsior et al., 2012b
rMEP	309-318 SAG1_EP2 109-118 SAG2_EP1 347-356 SAG3_EP2	Recombinant protein with His tag and Trx tag domains	IgG ELISA	108 total; 32 acute, 76 chronic	Sensitivity, 94.4% overall, 87.5% (acute-phase), 97.4% (chronic sera); specificity, 100%	Dai et al., 2012
			IgM ELISA	32 total	Sensitivity, 96.9%; specificity, 100%	
rMEP	309-318 SAG1_EP2 109-118 SAG2_EP1 347-356 SAG3_EP2	Recombinant protein with His tag and Trx tag domains	IgG ELISA	126 total pregnant women; 58 acute, 68 chronic	Sensitivity, 96.4% Overall; 25.9% (acute-phase), 97.1% (chronic sera); specificity, 98.7%	Dai et al., 2013
			IgM ELISA	58 total pregnant women	Sensitivity, 96.6%; specificity, 100%	
SAG2+GRA1+ROP1_L_	31-170 SAG2 26-190 GRA1 85-396 ROP1	Recombinant protein with His tag domain	IgG ELISA	172 total; 41 acute, 17 post-acute, 114 chronic	Sensitivity and specificity, 100%	Ferra et al., 2015
USM.TOXO1 (SAG1+GRA2+GRA7)	202-217 SAG1 67-82 SAG1 88-103 SAG1 61-76 GRA2 153-168 GRA2 28-42 GRA2 26-41 GRA7 199-214 GRA7 162-177 GRA7	Recombinant protein with His tag domain	IgG ELISA	157 total; 6 acute, 151 chronic	Sensitivity, 85.43%; specificity, 81.25%	Hajissa et al., 2015, 2017
SAG2+GRA1+ROP1+ AMA1N	31–170 SAG2 26–190 GRA1 85–396 ROP1 67–287 AMA1N	Recombinant protein with His tag domain	IgG ELISA IgM ELISA IgG avidity	192 total; 64 suspected acute, 128 chronic 124 total; 66 acute, 58 chronic 60 total; 29 suspected acute, 31 chronic	Sensitivity and specificity, 100% Sensitivity, 90.9%; specificity, 97.1% Sensitivity, 96.8%; specificity, 100%	Ferra et al., 2019
AMA1N+SAG2+GRA1 +ROP1	68–287 AMA1N 31–170 SAG2 26–190 GRA1 85–396 ROP1		IgG ELISA IgM ELISA IgG avidity		Sensitivity and specificity, 100% Sensitivity, 84.9%; specificity, 99% Sensitivity, 96.8%; specificity, 100%	
AMA1C+SAG2+GRA +ROP1	287–569 AMA1C 31–170 SAG2 26–190 GRA1 85–396 ROP1		IgG ELISA IgM ELISA IgG avidity		Sensitivity and specificity, 100% Sensitivity, 92.4%; specificity, 91.4% Sensitivity, 87.1%; specificity, 100%	
AMA1+SAG2+GRA1 +ROP1	68–569 AMA1 31–170 SAG2 26–190 GRA1 85–396 ROP1		IgG ELISA IgM ELISA IgG avidity		Sensitivity and specificity, 100% Sensitivity, 95.5%; specificity, 99% Sensitivity and specificity, 100%	

a *All recombinant proteins were produced in E. coli unless specified*.

All the studies mentioned above proposed that multi-epitopes and peptide proteins augment the test sensitivity, thus opening new possibilities in the serodiagnosis of *T. gondii* infection. The utilization of recombinant chimeras will not only enable the development of more precise and reliable diagnostic systems but will pave the way for the discovery of more promising tests that are capable of distinguishing early or recently acquired infections from the chronic ones.

## Conclusion

The diagnosis of *T. gondii* infection remains a big challenge. Currently, serological diagnosis plays a crucial role in the identification of *T. gondii* infections in both humans and other animals. But due to the inadequacy of accuracy and reliability of the current diagnostic tests brought about by the lack of standardization in the production of the *T. gondii* whole-cell lysates, the consideration of other diagnostic options is compelling. An increasing number of studies have presented the growing advantage of using recombinant proteins, used singly or in combination, or chimeric antigens for the serological detection of *T. gondii* infections, such as improving the standardization of detection kits (Kotresha and Noordin, 2010), reducing the costs of production (Holec-Gąsior, 2013) and increasing the probability of differentiating different phases of toxoplasmic infection (Sickinger et al., 2008). Nevertheless, there are still many issues to be resolved when using recombinant antigens as diagnostic antigens. The sensitivity of assays using recombinant antigens has been reported to be lower than that of assays using native antigens. Differences in cloning strategies, methods of recombinant protein purification, and criteria used in the data analyses could also lead to variance in sensitivities and specificities of diagnostic tests. These important points should be considered when interpreting the results of various reports (Holec-Gąsior, 2013; Zhang et al., 2016). Another concern is in the use of *E. coli* expression system where the recombinant antigens produced often lose their antigenic value due to incorrect folding, and some contamination with *E. coli* antigens in partially purified recombinant proteins are reported. One resolution to these problems is the production of recombinant proteins in eukaryotic expression systems. Studies have revealed that they have post-translational modification mechanisms that enable the production of recombinant proteins that have conformations almost identical to that of the native proteins. Moreover, they do not contain bacteria-derived contaminants, thus, avoiding cross-reactions with human sera (Biemans et al., 1998; Zhou et al., 2007; Lau et al., 2010, 2011, 2012; Chang et al., 2011; Thiruvengadam et al., 2011).

Meanwhile, bioinformatics has been useful in the analysis of biological data by employing various methods and technologies ranging from mathematics, statistics, and computer sciences to biology and medicine. It presents valuable results from the analysis of large amounts of raw data (Romano et al., 2011). One goal of bioinformatics is to effectively and promptly organize, analyze, and translate information from the genome, transcriptome, and/or proteome (Brusic and Flower, 2004). It has been extensively used to predict protein structures, functions, and other biological characteristics (Romano et al., 2011). Bioinformatics tools and online software have been extensively used to analyze gene and protein expression and predict the structure, immunogenicity, and general features of *T. gondii* proteins (Bai et al., 2012; Shaddel et al., 2018). Prediction of epitopes can show the pathogenesis and immune mechanisms of pathogens, thus vital data can be obtained to identify immunogenic peptides and useful for the development of diagnostic reagents and new vaccines (Bai et al., 2012). Likewise, approach to integrating multiple omics technologies—such as genomics, transcriptomics, proteomics, and metabolomics has been adapted to obtain a more comprehensive insight of the biology and disease for a better and holistic understanding of diagnosis and treatment protocols (Karczewski et al., 2018). In *T. gondii*, proteomic and genomic analyses and molecular modeling were utilized to characterize new rhoptry proteins of the ROP2 family to elucidate the specific roles of the proteins, especially in the early interaction with the host cell upon invasion (El Hajj et al., 2006). Molecular technologies including microsatellite analyses (Ajzenberg et al., 2010), multilocus sequence typing to recognize single nucleotide polymorphisms (Ajzenberg et al., 2002; Khan et al., 2005, 2007; Lehmann et al., 2006; Su et al., 2006) and polymorphic polypeptides from *T. gondii* antigens (Kong et al., 2003; Xiao et al., 2009) have been developed and applied for genotyping and serotyping *T. gondii* infections.

After consolidating all the findings presented by the different studies we reviewed in this paper, we conclude that the utility of recombinant proteins in the serodiagnosis of *T. gondii* infections is highly advantageous in improving the standardization of the tests and lessen their production costs. Combining several recombinant antigens with multiple immunodominant epitopes, as either a mixture or a chimeric product, significantly increases the probability of detecting *T. gondii* antibodies at different stages of the infection. The diagnosis of *T. gondii* infection continues to be challenging until relatively rapid and highly sensitive, and specific methods are developed. The direction now is to integrate genomic, transcriptomic, and proteomic technologies and multi-locus genotyping methods with molecular and bioinformatics tools for the advancement of detection methods utilizing these recombinant antigens. These new techniques help demonstrate the genetic diversity of *Toxoplasma* strains as well as the stage of infection, which would aid better in the diagnosis of toxoplasmic infection.

## Author Contributions

RY, AY, and YN have contributed equally during the conceptualization, writing, editing, and finalization of this review paper.

## Conflict of Interest

The authors declare that the research was conducted in the absence of any commercial or financial relationships that could be construed as a potential conflict of interest.
